# Missing pieces in decoding the brain oxytocin puzzle: Functional insights from mouse brain wiring diagrams

**DOI:** 10.3389/fnins.2022.1044736

**Published:** 2022-10-26

**Authors:** Steffy B. Manjila, Rebecca Betty, Yongsoo Kim

**Affiliations:** Department of Neural and Behavioral Sciences, The Pennsylvania State University, Hershey, PA, United States

**Keywords:** oxytocin, neural circuit, oxytocin receptor (OxtR), wiring diagram, mice

## Abstract

The hypothalamic neuropeptide, oxytocin (Oxt), has been the focus of research for decades due to its effects on body physiology, neural circuits, and various behaviors. Oxt elicits a multitude of actions mainly through its receptor, the Oxt receptor (OxtR). Despite past research to understand the central projections of Oxt neurons and OxtR- coupled signaling pathways in different brain areas, it remains unclear how this nonapeptide exhibits such pleiotropic effects while integrating external and internal information. Most reviews in the field either focus on neuroanatomy of the Oxt-OxtR system, or on the functional effects of Oxt in specific brain areas. Here, we provide a review by integrating brain wide connectivity of Oxt neurons and their downstream circuits with OxtR expression in mice. We categorize Oxt connected brain regions into three functional modules that regulate the internal state, somatic visceral, and cognitive response. Each module contains three neural circuits that process distinct behavioral effects. Broad innervations on functional circuits (e.g., basal ganglia for motor behavior) enable Oxt signaling to exert coordinated modulation in functionally inter-connected circuits. Moreover, Oxt acts as a neuromodulator of neuromodulations to broadly control the overall state of the brain. Lastly, we discuss the mismatch between Oxt projections and OxtR expression across various regions of the mouse brain. In summary, this review brings forth functional circuit-based analysis of Oxt connectivity across the whole brain in light of Oxt release and OxtR expression and provides a perspective guide to future studies.

## Introduction

Oxytocin (Oxt) was originally identified in 1906 as the primary molecule involved in parturition and lactation (Dale, 1906). Further studies over the past centenary identified this nonapeptide’s role in regulating multiple other behaviors both in the central and peripheral nervous systems (Lee et al., 2009; Jurek and Neumann, 2018; Lefevre et al., 2021; Wang et al., 2022). As further studies elucidate this molecule’s impact on brain and body systems, efforts to explore and establish the brain circuit level mechanisms of Oxt’s functional control have proliferated. However, most prior effort has been focused on specific behaviors with selected brain areas. Although these divide-and-conquer approaches have been critical in establishing the role of Oxt in individual brain regions, uniting these studies to gain a broad circuit-based understanding of the multitude of Oxt functions that modulate brain processing has been challenging. Here, we provide an integrated view to link Oxt’s influence on connected neural circuits with the different behaviors Oxt evokes. Thus, we aim to present new insights into the relationship between Oxt’s anatomy and function.

## Oxytocin: A historical perspective

Sir Henry H Dale, a British physiologist, initially discovered that extracts from pituitary glands (from oxen) when administered intravenously facilitated contractions of the uterus (Dale, 1906). In 1909, gynecologist William Blair Bell performed investigations that proved that pituitary extracts facilitated uterine contractions and helped with fetal delivery (Bell, 1909). In the following year, another study demonstrated a role for the same extract in milk ejection in animals (Ott and Scott, 1910). With the advancement in biochemical methods, the chemical structure of the molecule Oxt was identified in 1953 (Tuppy, 1953) and synthesized by Vincent du Vigneaud in 1954 (du Vigneaud et al., 1954). Further studies in the following years identified the neurons that produced Oxt (Bargmann and Scharrer, 1951) and the receptor that Oxt binds to (Gimpl and Fahrenholz, 2001). For more details, we refer to previous papers describing the long history of Oxt starting as a facilitator of parturition to how it reached its current status of a social hormone that functions within the brain (Lee et al., 2009; Carson et al., 2013; Froemke and Carcea, 2017; Jurek and Neumann, 2018).

## Neuroanatomy and functions of Oxt neurons

Oxt is synthesized mainly in the paraventricular hypothalamus (PVH) and the supra optic nucleus (SO) along with a smaller number of neurons in the accessory nuclei of the hypothalamus and extended amygdala (Freda et al., 2022; Son et al., 2022). In addition, our recent study identified a dense cluster of Oxt neurons in the tuberal nucleus area (TU) of the hypothalamus in the mouse brain (Son et al., 2022). Oxt functions mainly through a single Oxt receptor (OxtR) that is expressed on the target tissues throughout the body, and enriched in most parts of the brain (Gimpl and Fahrenholz, 2001; Assinder, 2022). Oxt elicits its actions through OxtRs in the peripheral system to regulate gastric motility, heart rate, breathing, vasodilation and regulation of blood glucose levels via insulin (Filipov and Kasakov, 1978; Higa et al., 2002; Mack et al., 2002; Welch et al., 2014; Pozo and Claret, 2018). In the central nervous system, Oxt is known to regulate multiple aspects of social behaviors including recognition, memory, pair bonding and maternal bonding (Nagasawa et al., 2012; Oettl et al., 2016; Ophir, 2017; Raam et al., 2017). Oxt is also known to exert its effects on sleep, reward systems, and several aspects of sensory systems ranging from olfaction and taste to vision and hearing (Hung et al., 2017; Grinevich and Stoop, 2018; Raymond et al., 2021). Not surprisingly, dysfunctional Oxt signaling has been implicated in several neurological disorders, most notably autism spectrum disorder (ASD) and even Alzheimer’s Disease (Ishunina and Swaab, 2002; Yamasue and Domes, 2018; Szczepanska-Sadowska et al., 2022).

## Mismatch between Oxt projections and OxtR: Implications on Oxt release mechanisms

The widespread expression of OxtRs compared to relatively fewer areas of Oxt neuronal projections throughout the brain has been a puzzling problem in the field over the past few decades. This led to multiple hypotheses on the release mechanisms of Oxt (Chini et al., 2017). But recent literature suggests most OxtR enriched areas contain at least sparse projections in the rat brain (Grinevich et al., 2016). In our recent publication, we quantified and compared Oxt projections and OxtR density in the whole mouse brain. Except for regions within the thalamus and medulla, our analysis revealed no significant quantitative correlation between Oxt projection and OxtR density in most brain areas (Son et al., 2022). For example, most regions within the cerebral cortex of the mouse brain are enriched with OxtRs, but the Oxt projection fibers are sparse. However, Oxt modulates multiple behaviors by its effect in the cerebral cortex. For example, Oxt reduces GABAergic inhibition in cortical areas like the auditory areas (AUD) and piriform areas (PIR) to enhance the auditory and olfactory responses (Marlin et al., 2015; Mitre et al., 2016). Another example is the main olfactory bulb (MOB) which has abundant OxtRs but very minimal to null Oxt projections. Oxt addition to the MOB resulted in enhanced neuronal activity. Conversely, many hindbrain areas (hindbrain reticular nuclei) have Oxt projections and little to no OxtRs in the mouse brain. This suggests that Oxt signaling mechanisms could be different from the canonical synaptic transmission with direct projection. For example, Oxt uptake to the MOB is postulated to occur via a trans ventricular pathway [cerebrospinal fluid (CSF)-Subarachnoid-lymphatic system] (Veening et al., 2010). This is a very feasible pathway considering the distance between MOB to Oxt producing neurons, and the presence of Oxt fibers along the sides of the 3*^rd^* ventricle (Son et al., 2022). Further studies are required to confirm ventricular release of Oxt and the brain regions that uptake Oxt from the trans ventricular pathway.

Another hypothesis postulates that Oxt released into the blood stream is further transported to the CSF through the receptor for advanced glycation end-products (RAGE) (Yamamoto et al., 2019). This hypothesis is relevant for the uptake of externally administered Oxt to reach the brain, whether or not endogenous Oxt follows this pathway is still debated in the field. To add to the complexity, Oxt is also released from somata and dendrites (Ludwig, 1998). This release is mostly evoked by other peptides. Oxt itself can exert a feed forward activation of PVH and SO Oxt producing neurons, in turn resulting in further Oxt release (Moos et al., 1984; Neumann et al., 1996; Hirasawa et al., 2004). Oxt is also released *en passant*, a synapse independent mechanism, resulting in the release of a small number of vesicles at a specific target region. This is a diffusion based slow release of Oxt resulting in 60-90s delayed responses (Chini et al., 2017). Site specific dendritic release explains the mismatch between CSF concentrations and site specific concentrations of Oxt (Ludwig and Leng, 2006). It is also believed that dendritic release of Oxt causes excitation of nearby (100 um) neurons of a different nature, thus mediating population level cross talk between neurons at a specific site (Son et al., 2013). All these evidence cumulatively suggest site specific release as well as diffusion based and CSF based transport of Oxt, that can explain the mismatch between Oxt fibers and OxtRs. Hence, it is important to carefully analyze the brain areas that are enriched with projection fibers to understand the site-specific release associated functions of Oxt.

In subsequent sections, we link neural connectivity and the diverse functional effects of Oxt with OxtR expression and broadly classify brain regions with Oxt connections into three major functional modules; **internal state control, somatic visceral control and cognitive control** (Son et al., 2022). These modules are clustered based on known functions of regions (All abbreviations can be found in Table 1). Each module is further divided into three inter-connected circuits to process distinct information. Here, we will review individual circuits with their function and contribution of Oxt signaling to shape signal processing. Moreover, we compare whole brain Oxt projections with OxtR density and highlight the main areas of apparent discrepancies to discuss the functional relevance of Oxt in these mismatched areas.

**TABLE 1:** List of abbreviations.

Abbreviations	Full names
AI	agranular insular area
AOB	accessory olfactory bulb
AON	anterior olfactory nucleus
ARH	arcuate hypothalamic nucleus
AUD	auditory areas
BF	basal forebrain
BMA	basomedial amygdalar nucleus
BST	bed nucleus of stria terminalis
CEA	central amygdalar nucleus
CP	caudoputamen
CU	cuneate nucleus
DMH	dorsomedial nucleus of the hypothalamus
DMX	dorsal motor nucleus of the vagus nerve
DR	dorsal raphe nucleus
GPe	globus pallidus, external segment
GPi	globus pallidus, internal segment
GU	gustatory areas
IC	inferior colliculus
LC	locus ceruleus
LG	lateral geniculate complex
LHA	lateral hypothalamic area
LPO	lateral preoptic area
LS	lateral septal nucleus
MEPO	median preoptic nucleus
MG	medial geniculate complex
MO	somatomotor areas
MOB	main olfactory bulb
mPFC	medial prefrontal cortex
MY	Medulla
NTS	nucleus of the solitary tract
OT	olfactory tubercle
PAG	periaqueductal gray
PB	parabrachial nucleus
PGRN	paragigantocellular reticular nucleus
PIR	piriform area
PPN	pedunculopontine nucleus
PRN	pontine reticular nucleus
PRT	pretectal region
PVH	paraventricular hypothalamic nucleus
RM	nucleus raphe magnus
SC	superior colliculus
SCH	suprachiasmatic nucleus
SLC	subceruleus nucleus
SNr	substantia nigra, reticular part
SOC	superior olivary complex
SS	somatosensory areas
STN	subthalamic nucleus
TH	Thalamus
VII	facial motor nucleus
VIS	visual areas
VMH	ventromedial hypothalamic nucleus
VPL	ventral posterolateral nucleus of the thalamus
VPM	ventral posteromedial nucleus of the thalamus
VPMpc	ventral posterolateral nucleus of the thalamus, parvicellular part

## Oxytocin associated circuitry in internal state control

**FIGURE 1:**
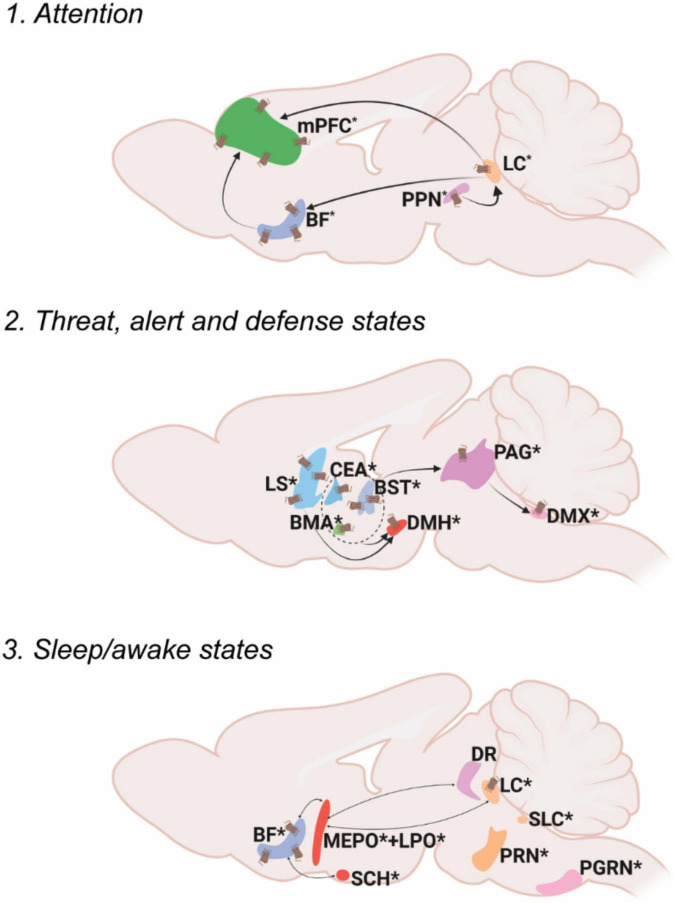
Oxt associated circuitry in internal state control. Pathways depicting three different functional circuits: 1. Attention, 2. Threat, alert and defense states, 3. Sleep/awake states. * denotes the presence of Oxt projection fiber in the specified area. Receptor symbol depicts presence of OxtR on the area mentioned. For abbreviations, refer Table 1.

Internal state can be defined as the combination of cellular and metabolic activities that modify the sensory information representation and the communication between body and brain (Kanwal et al., 2021). The main characteristic that delineates the internal state node is the regulation of baseline neural conditions that attenuate other responses within the body. This means that while internal state control does not regulate fast responses to stimuli, this control can create a predisposition to certain responses. As such, alterations to the internal state have broad influence on levels of attention, alertness and aggression, sleep, arousal and more (Figure 1).

### Internal state module: Attention

Attention can be defined as the cognitive and behavioral processes that allow one to preferentially select relevant information present in the environment as well as avoid irrelevant information (Sarter et al., 2001). Most of the brain regions in the attention circuit receive Oxt projection fibers and express OxtRs to modulate the behavioral response. The two major attention systems in the brain include the cholinergic system of the basal forebrain (BF) and norepinephrine system of the locus ceruleus (LC).

Multiple areas of the BF- diagonal band nucleus (NDB), substantia innominata (SI), magnocellular nucleus (MA) and medial septal nucleus (MS) release acetylcholine upon receiving an attention relevant cue in the rat brain (Záborszky et al., 2018; Laszlovszky et al., 2020). Although there is no direct evidence to prove that Oxt modulates attention in rodent studies, hypothalamic Oxt neurons send their long-range projections to both the BF and the LC (Son et al., 2022). Moreover, the BF is an area that is also enriched in OxtRs, suggesting the possibility of Oxt modulation of the BF attention circuit (Newmaster et al., 2020). In human postmortem samples of Autism Spectrum Disorder (ASD) patients, Oxt binding to OxtR was found to be reduced in the Nucleus Basalis of Meynert (NBM), a subset of neurons in the SI, suggesting the role of Oxt in disease progression of ASD (Freeman et al., 2018). Recent clinical studies also point toward the role of Oxt in modulation of social attention. Intranasal Oxt administration improved socially directed gaze and increased attention allocation to familiar faces (Freeman et al., 2018; Marsh et al., 2021). Moreover, intranasal Oxt enhanced the attention allocation to faces in autistic individuals (Kanat et al., 2017).

The LC sends adrenergic projections to the medial prefrontal cortex (mPFC), thalamus (TH), BF and many other brain areas (Sara, 2009; Chandler et al., 2019). Oxt is known to increase LC adrenergic receptor responsiveness and thus enhance salience of received cues in rats (Petersson et al., 1998). Optogenetic activation of PVH-Oxt neurons in rat brain resulted in downstream activation of LC-noradrenaline followed by an enhanced attention to novel objects suggesting that Oxt directly regulates attention by modulating LC responses (Wang et al., 2021). The LC also receives monosynaptic inputs from another attention relevant area called the pedunculopontine nucleus (PPN), an area with abundant Oxt neuronal fibers. This suggests multiple levels at which Oxt can modulate attention responses, at either the PPN or LC. Oxt at the PPN can modulate adrenergic response from the LC, thus acting as a neuromodulator of another neuromodulator.

There are several other brain areas along with the BF and LC, that are relevant in the attention circuit. For example, the claustrum (CLA) in primates is an area relevant for top-down control of attention with its extensive connections to the neocortex (Mathur, 2014; Atlan et al., 2018; Zingg et al., 2018). The mouse CLA predicts upcoming movement in between intertrial intervals (Chevée et al., 2022). Interestingly, Oxt neurons project to the CLA-Endopiriform complex in the mouse brain, an area with high density of OxtRs, the functional relevance of which has yet to be explored (Dubois-Dauphin et al., 1992; Newmaster et al., 2020; Biggs and Hammock, 2022).

### Internal state module: Threat, alert, and defense states

Fear is a combined response constituting physiological (cardiovascular, respiratory) and behavioral (freeze, fright, startle) counterparts experienced due to an exposure to possible threats that can affect one’s survival (Olivera-Pasilio and Dabrowska, 2020). The brain receives external threat (predator) cues to the limbic forebrain and extended amygdala (bed nuclei of stria terminalis, BST; lateral amygdalar nucleus, LA; medial amygdalar nucleus, MEA and central amygdalar nucleus, CEA). Once a threat cue is received, the immediate response is arousal- which activates the downstream defense cascade. Arousal is the state of being awake and alert- mostly regulated by the hypothalamus (ventromedial hypothalamic nucleus, VMH and dorsal premammillary nucleus, PMd)- by increasing the tone in the sympathetic (visceromotor) and autonomic (striated muscles) nervous systems. The next step in the fear response cascade invokes the flight or fight response, resulting in specific motor patterns of flight or fight: attack, run, freeze etc. (Kozlowska et al., 2015). The skeletal muscle activation for fight and attack is mediated through the periaqueductal gray (PAG) whereas visceromotor output (for example, increasing cardiac output) is mediated via the dorsal motor nucleus of the vagus nerve (DMX) (Kozlowska et al., 2015; Deng et al., 2016). Alternatively, the freezing response may occur, which puts on hold the flight or fight response- activated mainly through the vagal pathway from the DMX by inhibiting sympathetic activation (Roelofs, 2017; Roelofs and Dayan, 2022).

Oxt neurons send projections to all these areas, controlling many aspects of fear response. For example, Oxt released from the PVH to the extended amygdala (the CEA and the BST), is critical for regulating fear/threat responses (Duque-Wilckens et al., 2020; Olivera-Pasilio and Dabrowska, 2020). Oxt in the CEA reduces cued fear whereas in the BST, Oxt facilitates cued fear responses and avoids diffuse threats (Knobloch et al., 2012; Francesconi et al., 2021). Oxt acts mainly by the strong binding of Oxt to OxtRs in the BST and this also creates an anxiogenic effect (Martinon et al., 2019). The brain processes contextual threat cues (predator exposure) slightly different than other threat cues (predator odor). The lateral septum (LS) processes contextual cues and sends responses to the hypothalamus (Cezario et al., 2008). Oxt signaling in the LS regulates social fear during lactation (Menon et al., 2018). Altogether, Oxt can exert differential effects on multiple brain areas after gauging the input threat due to its vast connectivity to interconnected brain areas in the fear circuit.

### Internal state module: Sleep/awake states

Sleep can be defined as a fast reversible state of immobility accompanied by reduced neurophysiological and behavioral responses to environmental stimuli (Raymond et al., 2021). Sleep is mainly categorized into REM (rapid eye movement) sleep and NREM (non-rapid eye movement) sleep. Although the complete sleep circuit is unclear, main brain areas and molecules/neurotransmitters that mediate REM and NREM sleep have been identified. The REM/NREM sleep transitions regulate only the short stretches of sleep as opposed to the whole night’s sleep regulated through different brain centers (Scammell et al., 2017).

One of the earliest identified sleep promoting areas is the preoptic area (Von Economo, 1930). The NREM active lateral preoptic area (LPO) and median preoptic nucleus (MEPO) neurons are GABAergic and inhibit the attention/alert/wake promoting centers consisting of the BF, dorsal raphe (DR), and LC. These neurons also produce galanin, a neuropeptide very highly associated with sleep regulation (Ma et al., 2019). All the attention/alert centers are also reciprocally connected to the ventrolateral preoptic nucleus (VLPO) to inhibit galanin neurons during awake states. The brain areas active during REM sleep include pontine areas- the subceruleus nucleus (SLC), the pontine reticular formation (PRN), and the paragigantocellular nucleus (PGRN). Most of the sleep active neurons are reciprocally connected with inhibitory neurons in the alert/attention centers and selective activation of one group determines the sleep/wake state (Scammell et al., 2017). The transition between sleep to wake states based on day-night cycles is majorly controlled by the circadian system/biological clock possessed by most animals. The central pacemaker that regulates circadian rhythm is the suprachiasmatic nucleus (SCH). SCH activity oscillates with day-night cycles based on the transcriptional regulation of a group of circadian genes (Eban-Rothschild et al., 2018).

While Oxt has been implicated in this system, Oxt’s role in sleep regulation remains relatively understudied. Oxt neurons project to all the previously mentioned major nodes that control REM, NREM and circadian aspects of sleep. Despite the lack of evidence for the exact mechanism of sleep regulation by Oxt, there are several clinical studies implicating better quality sleep with increased Oxt levels in humans. For instance, increased endogenous Oxt in postpartum women facilitates sleep (Comasco et al., 2016). Oxt administration in obstructive sleep apnea patients shortened the duration of apnea (Jain et al., 2017). Although evidence suggests that Oxt improves sleep quality, this could also be attributed to the anxiolytic abilities of Oxt indirectly enhancing sleep (Comasco et al., 2016). However, Oxt levels in sleep deprived Syrian Hamsters were increased in the SO suggesting an endogenous pathway that activates Oxt to regulate sleep. In male rats, intracerebral Oxt administration under stress free conditions enhanced sleep (Lancel et al., 2003). Since Oxt is connected to the main sleep regulatory brain areas and clinical studies show positive sleep regulation, further research understanding the mechanisms by which Oxt modulates sleep could help improve our knowledge of disorders like insomnia, sleep apnea, narcolepsy etc. along with other psychological disorders in which sleep is perturbed.

## Oxytocin regulation of somatic/visceral control

**FIGURE 2:**
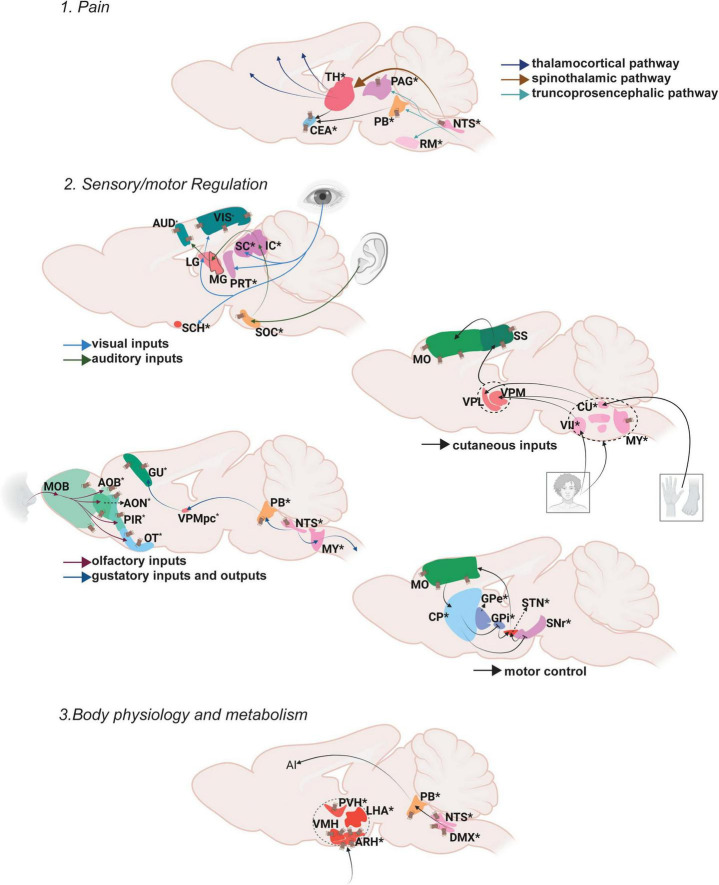
Oxt regulation of somatic/visceral Control. Pathways depicting three different functional nodules.1. Pain, 2. Sensory/motor regulation, 3. Body physiology and metabolism. For further details refer, Figure 1 legend. For abbreviations, refer Table 1.

Oxt in the somatic/visceral control module share abilities that regulate a host of homeostatic activities and can be further subdivided into three groups: pain controlling areas, areas involved in sensory motor regulation and areas regulating body physiology and metabolism (Figure 2).

### Somatic/visceral module: Pain

Pain is a distressing sensation and an emotional experience often associated with either actual or potential tissue damage, with the major intention of starting the body’s defense mechanisms to react toward the causal stimuli and thus prevent further tissue damage. The ascending pathway carries pain information from the peripheral (dorsal root ganglion) to the central nervous system (PNS and CNS, respectively) while the descending pain pathway brings responses from CNS to the peripheral reflex organs (Yam et al., 2018).

Ascending pain pathways include spinothalamic pathways that relay pain information from the spinal cord to different thalamic areas followed by thalamocortical pathways which relay information from thalamus to the cortical centers (e.g., somatosensory cortex) that process different aspects of pain perception. The medial thalamus (mediodorsal nucleus of thalamus, MD; parafascicular nucleus, PF; central lateral nucleus of the thalamus, CL) sends the emotional and motor related information to the mPFC (e.g., anterior cingulate cortex, ACC). The lateral thalamus (ventral posterolateral nucleus of the thalamus, VPL) conveys sensory pain information to the somatosensory cortex (SS), and the posterior thalamus (lateral posterior nucleus of the thalamus, LP; suprageniculate nucleus, SGN) relays pain perception and intensity information to the posterior insular cortex (Purves et al., 2001; Khera and Rangasamy, 2021; Mercer Lindsay et al., 2021). Another pathway that carries pain information is the truncoprosencephalic projections that include parabrachial nucleus (PB) connections to the amygdala and further to the cingulate and the insular cortices (Barik and Chesler, 2020; Kuner and Kuner, 2021). The ascending information also passes through the PAG in the midbrain and rostroventral medulla (RVM) in the hindbrain where the PAG integrates the descending output to the spinal cord (Basbaum et al., 2009). Overall, a vast majority of brain areas especially in the thalamus regulate various aspects of pain processing.

Oxt exerts its effects on both ascending and descending brain regions that modulate pain. Oxt neurons send projections to multiple thalamic areas (Medial: subparafascicular area, SPA; parafascicular nucleus, PF; lateral peripeduncular nucleus, PP) to regulate the ascending pain pathway. Oxt neurons also send projections to the PB and various amygdalar nuclei to modulate the pain inputs. In the descending pathway, Oxt neurons project to the nucleus raphe magnus (RM) to further modulate pain responses (Lefevre et al., 2021; Son et al., 2022). Even though Oxt innervates only a few areas that regulate pain, it exerts an effect on brain regions of both the ascending and descending pathway resulting in multi-level regulation of pain.

Oxt is known to have analgesic effects, both from preclinical and clinical studies. In rats, partial sciatic nerve ligation induced pain results in increased Oxt synthesis (Nishimura et al., 2019). Moreover, Oxt modulates the GABAergic tone of the insular cortex and thus exerts an analgesic effect in mice (Gamal-Eltrabily et al., 2020). In clinical studies, intranasal Oxt administration attenuated pain in patients with lower back pain, and in women with chronic pelvic pain (Schneider et al., 2020; Flynn et al., 2021). These data strongly suggest analgesic effects of Oxt, but further investigation is required to understand the mechanism by which Oxt elicits these effects.

### Somatic/visceral module: Sensory/motor regulation

Sensorimotor integration is defined as the ability to integrate different sources of sensory stimuli in the central nervous system and transform this into goal directed motor functions (Machado et al., 2010). The motor system is controlled mainly via the basal ganglia circuit that functions through motor activity in the whole body, mainly the forelimbs and hindlimbs.

The visual inputs from the retina reach the lateral geniculate nucleus (LG) in the thalamus from which the information is sent to the visual cortex (VIS). Visual information is passed on to the suprachiamatic nucleus (SCH) of the hypothalamus from the retina to direct attention to the visual stimuli (Peirson et al., 2018). The pretectum (PRT) also receives visual information from the retina and SCH to modulate visuomotor behaviors (Levine and Schwartz, 2020). Oxt projects to the VIS, PRT and SCH (Son et al., 2022). The superior colliculus (SC), a midbrain area extensively innervated by Oxt neurons, also receives visual input from retina as well as processed information from the VIS especially while tracking objects (Zubricky and Das, 2022). There is no direct evidence to prove the effect of Oxt on vision. Social attention measured by changes in eye gazes are facilitated upon intranasal Oxt administration (Freeman et al., 2018). Whether this can be attributed to effects of Oxt on vision or attention is not clear.

Auditory inputs received via the cochlea are sent to the superior olivary nucleus (SOC) followed by the inferior colliculus (IC). The IC is the relay station where ascending and descending auditory pathways converge. From the IC, auditory signals are passed to the AUD through the medial geniculate nucleus (MG) (Morgan et al., 2020). The posterior intralaminar thalamic nucleus (PIL) functions as an integrator of auditory information before it is passed to the AUD (Smith et al., 2019). Oxt neuronal projections reach all the relevant brain regions for auditory processing (SOC, IC, PIL, AUD). Studies in rodents demonstrated that Oxt can induce plasticity in AUD which help facilitate parent responses to infant cries (Schiavo et al., 2020). A recent study identified that the PIL responds to pup calls to mediate maternal behavior (Valtcheva et al., 2021). Clinical studies so far have only correlative evidence suggesting Oxt regulates auditory responses. In humans, listening to their mother’s voice decreased infant pain scores while increasing Oxt levels above baseline (Filippa et al., 2021). Intranasal Oxt administration in ASD patients has significantly improved the ability to select speech sounds from background noise (Lin et al., 2014; Yoshimura et al., 2018).

Olfactory inputs arise from the olfactory epithelia and reach the main MOB. From the MOB, it projects to the taenia tecta (TT), anterior olfactory nucleus (AON), PIR, olfactory tubercle (OT) and entorhinal cortex (ENT). Vomeronasal inputs that carry pheromone information also start from the olfactory epithelium, but flow to the accessory olfactory bulb (AOB) and further to the bed nucleus of stria terminalis (BST), nucleus of the lateral olfactory tract (NLOT) and various amygdaloid nuclei. Processed olfactory information is passed to multiple brain regions depending on the action to be performed (De Castro, 2009; Canavan et al., 2011). Despite the high density of OxtRs, Oxt neurons have limited projection to olfactory brain regions (Newmaster et al., 2020; Grinevich and Neumann, 2021; Son et al., 2022). However, stimulation of Oxt neurons as well as injection of Oxt in the MOB resulted in increased neuronal activity (Yu et al., 1996). This increased activity is attributed to the binding of Oxt to OxtR in the MOB (Vaccari et al., 1998). These data suggest that Oxt can powerfully modulate olfactory signal processing in the olfactory bulb even without direct projection. There is also evidence that OxtR activation in the MOB increases the signal to noise ratio for odorant evoked responses (Oettl et al., 2016). These suggest a highly sensitive and projection fiber independent activity of Oxt on OxtRs.

Taste inputs received through the taste receptors reach the brain through the nucleus of the solitary tract (NTS). From the NTS, there are three distinct pathways that relay taste information: the reflex pathway for either enhancing or reducing ingestion, the lemniscal pathway for taste perception and discrimination, and the visceral-limbic pathway mostly for regulating homeostatic and motivational states. The reflex pathway involves NTS signals conveyed to medullary and reticular formations that further innervate the cranial motor nuclei (Martin and Sollars, 2017). The lemniscal pathway from the NTS relays through the PB and further to the gustatory cortex (GU) via the ventral posterior medial nucleus of the thalamus (VPMpc). This pathway also transitions the sensation from somatosensory lingual to gustatory representation in the brain through the signals received in the agranular insular cortex (Stapleton et al., 2006). The visceral-limbic system connects the NTS to BF areas, the lateral hypothalamus (LH), the BST and the amygdala. Among these areas that control multiple facets of taste perception, discrimination and memory, Oxt innervates the NTS, PB, GU and BST. Thus, Oxt could modulate reflex, leminscal and visceral-limbic gustatory pathways by either exerting its effect on one specific node or on multiple nodes of these pathways. So far there is no direct evidence for Oxt controlling taste perception and discrimination.

The cutaneous afferents from both the forelimbs and the hindlimbs reach the thalamus either through direct spinothalamic pathways or from the spinal cord via the cuneate nucleus (CU). Tactile information is processed in different areas of the ventral posterior complex of the thalamus: the ventral posterolateral nucleus of the thalamus (VPL) –from the foot and hand (dorsal root ganglion-cuneate nucleus-RVM) and the ventral posteromedial thalamus (VPM) -from the face (through spinal trigeminal nucleus-VII). From the thalamus this information is passed to the somatosensory cortex that then delivers the processed information to the motor cortex (Qi et al., 2014a). Oxt projections reach areas in the medulla- spinal trigeminal nucleus (spinal nucleus of the trigeminal caudal part, SPVC and interpolar part, SPVI -sensory associated) that receive temperature, touch and pain information from the face through the facial motor nucleus (VII -motor related) and reticular formation (gigantocellular reticular nucleus, GRN; parvicellular reticular nucleus, PARN; lateral reticular nucleus, LRN) responsible for motor movements of head and face (Patel and Das, 2021). Despite Oxt projections, OxtRs are present in multiple somatosensory control areas within the mouse brain including the somatosensory and motor cortices extensively (Newmaster et al., 2020). It is yet to be explored whether Oxt directly affects tactile information processing.

The integration of visual, auditory and somatosensory inputs occurs in the multisensory midbrain, the superior colliculus (SC), which also receives direct projections from Oxt neurons. The heavy inputs from the SC to Oxt neurons transmit visual information required for learning of pup retrieval in virgin female mice, a process that requires integration of multiple inputs (Carcea et al., 2021). Although OxtRs are not reported so far in the mouse brain SC, there is a report suggesting presence of OxtRs in the superficial layers of the SC in primates (rhesus monkeys) (Freeman et al., 2014; Newmaster et al., 2020).

Motor control is a well-studied area which mainly involves the basal ganglia circuit. The dorsal striatum (caudatoputamen, CP), the globus pallidus (GP), and the subthalamic nuclei (STN) as major stations of the basal ganglia receive motor inputs from cortical and sub cortical areas. There are three main pathways that control different aspects of locomotion. The direct pathway relays signal from the motor cortex that results in active motor responses. The motor cortex sends excitatory inputs to the CP, which in turn results in inhibition of the globus pallidus internus (GPi) and substantia nigra pars reticulata (SNr), activating the thalamus and resulting in motor activity. The indirect pathway mediates inhibitory outflow and thus reduces the motor activity. Inputs from the motor cortex reach the globus pallidus externa (GPe) that inhibits the STN, resulting in the activation of the GPi and SNr, causing inhibition of motor activity. The hyperdirect pathway is a direct relay signal from the motor cortex to STN inhibiting ongoing motor activity (Schroll and Hamker, 2013). Oxt projects to all the areas in the mouse brain motor circuit including the CP, GP, SN and STN and has the capability to modulate all three motor pathways (Son et al., 2022). For example, locomotor activity was reduced upon Oxt addition or OxtR inhibition in the SNc. Oxt modulates this locomotor activity by influencing the nigral glutamatergic tone in rats (Angioni et al., 2016).

### Somatic/visceral module: Body physiology and metabolism

Body physiology is the branch of biology that deals with regulating the normal function of organs. Metabolism can be defined as the sum total of all reactions within the body that converts food to energy (Sánchez López de Nava and Raja, 2022). Major brain areas that integrate glucose sensing to regulate metabolism include the paraventricular hypothalamic nucleus (PVH), ventromedial hypothalamic nucleus (VMH), lateral hypothalamic area (LHA) and arcuate hypothalamic nucleus (ARH) (McCormack et al., 2020). There are multiple reviews that extensively explain the role of Oxt in energy metabolism (Cezario et al., 2008; Song et al., 2014; Lawson, 2017; Ding et al., 2019; Onaka and Takayanagi, 2019; McCormack et al., 2020; Kerem and Lawson, 2021). For instance, OxtRs are enriched in the ARH and these neurons control satiety (Fenselau et al., 2017). Moreover, Oxt in the NTS regulates food seeking and motivation (Wald et al., 2020).

Body physiology and metabolism is not only regulated by nutrient/glucose uptake, but also by fluid intake and homeostasis. Fluid intake and regulation is maintained mainly through the projections from NTS to PB and downstream areas- the CEA, MEPO, vascular organ of lamina terminalis (OV) and anteroventral periventricular nucleus (AVPV) (Zimmerman et al., 2017). Fluid intake is also directly regulated via Oxt through its projections to the PB, specifically by neurons that express OxtRs (Ryan et al., 2017). More evidence for Oxt regulation of fluid intake can be found at Arletti et al., 1990; Ryan, 2018

Cardiovascular activity is also regulated by Oxt that is released from the brain (Petersson, 2002). The NTS and DMX are very closely situated brain regions that regulate cardiovascular activity. They both control baroreflex control of the heart. Oxt inputs via neuronal terminals present at the solitary-vagal complex increase vagus outflow and thus result in a slower heart rate (Higa et al., 2002; Karelina and Norman, 2009). Both of these areas are also enriched with OxtRs in rodents (Barberis and Tribollet, 1996). There are several reviews that explain the cardiovascular control of Oxt in detail (Gutkowska and Jankowski, 2012; Jankowski et al., 2020; Szczepanska-Sadowska et al., 2021; Wang et al., 2022).

## Oxytocin regulation of cognitive control

**FIGURE 3:**
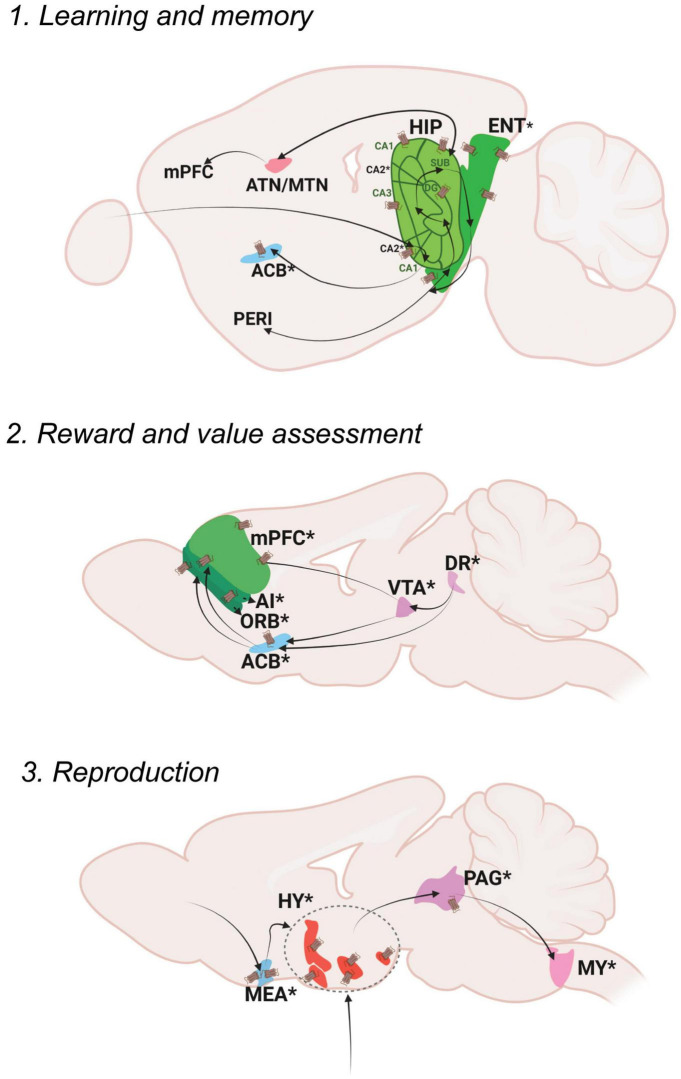
Oxt regulation of Cognitive Control. Pathways depicting three different functional nodules: 1. Learning and memory, 2. Reward and value assessment, 3. Reproduction. For further details refer, Figure 1 legend. For abbreviations, refer Table 1.

Cognitive control refers to the selection of emotions, behaviors and thoughts based on social context and current requirements along with avoiding inappropriate actions (Miller and Cohen, 2001). The cognitive control node involves Oxt’s regulation of mental processes—from learning and memory to reward and value assessment—giving rise to a wide array of social and sexual behaviors (Figure 3).

### Cognitive control module: Learning and memory

Memory is defined as the process of encoding, storing and retrieving information (Squire, 2009). Memory can be subdivided into explicit memory, implicit memory and working memory.

Explicit memories include events that happened in the past (episodic) as well as general information and facts (semantic). Episodic memory mainly functions through neocortex (perirhinal area; PERI)-entorhinal area (ENT) -hippocampal circuits (dentate gyrus; DG, areas within Ammon’s horn; CA3, CA1, subiculum; SUB) (Bird and Burgess, 2008). The hippocampus is also connected to the retro splenial cortex (RSP) and thalamic nuclei (anterior thalamic nucleus; ATN and midline thalamic nuclus; MTN) to further facilitate the process of memory storage and retrieval (Ophir, 2017).

Memory modulation by Oxt is studied specifically in the context of social memory (Lin and Hsu, 2018). OxtRs are enriched in the CA2 area of the mouse brain, especially in the pyramidal neurons (Young and Song, 2020). Oxt fibers that reach CA2 neurons enhance the rate of signal transfer in the hippocampus by creating burst firing, which in turn results in short term plasticity and feedforward activation on CA1 neurons (Tirko et al., 2018). OxtRs in the CA2/3 area are also required for discrimination of social stimuli, but not required for non-social stimuli (Raam et al., 2017). Impaired social recognition was also observed in a CA2 specific OxtR knockout mouse (Lin et al., 2018). The ENT receives sparse projection from Oxt neurons and is enriched with OxtRs. Oxt inputs are required for ENT to CA2 plasticity that regulates long term social recognition memory in mice (Lin et al., 2018). The supramammillary nucleus (SUM) that has OxtRs, is also required for the formation of short term and long term social recognition memory (Rajamani et al., 2022). These are the major areas that show relevant social memory effects with perturbed Oxt signaling. Oxt fibers also reach other memory relevant thalamic nuclei like the ATN and nucleus of reuniens (RE).

### Cognitive control module: Reward and value assessment

Reward is an act of pleasant or positive affective experience (White, 2011). The main reward system in the brain is the dopaminergic mesolimbic pathway. Once a stimulus that indicates reward is received, dopamine is released from the ventral tegmental area (VTA) to the nucleus accumbens (ACB). From the VTA to ACB forms the mesolimbic pathway and the VTA to PFC forms the meso-cortical circuits, the most relevant pathways for reward. The serotonergic dorsal nucleus raphe (DR) neurons project on the VTA to modulate reward responses (Qi et al., 2014b). Thus, dopaminergic VTA and serotonergic DR together function to shape the reward responses that are sent either to the ACB or different cortical areas- the mPFC areas- the infralimbic area (ILA) and anterior cingulate area (ACA) along with the orbital area (ORB) and agranular insular area (AI). The DR also projects to the ACB directly to modulate reward responses.

Social reward is mainly regulated by Oxt’s effect on reward circuitry. Oxt released in the VTA promotes prosocial behaviors. Optogenetic activation of Oxt-PVH neurons resulted in activation of reward specific VTA dopamine neurons that project to the ACB. This circuit is necessary to elicit social reward (Hung et al., 2017; Borland et al., 2019). DR serotonergic inputs to the OxtR positive ACB are also required for social reward. Ablation of OxtRs in the ACB resulted in impaired social reward processing (Dölen et al., 2013). Pair bonding observed in female prairie voles is also driven through Oxt in the ACB along with dopamine receptor 2 (Liu and Wang, 2003; Borie et al., 2022). There were reports suggesting changes in OxtR levels leading to pair bonding in prairie voles (Ophir et al., 2012; King et al., 2016). A more recent study postulates that OxtR are not required for social attachment in prairie voles using a CRISPR knockout model of OxtR, that has to be further evaluated in the light of previous literature (Berendzen et al., 2022). Oxt present in the ACB during a critical period during development establishes social reward learning (Nardou et al., 2019). Similar reward associated behaviors are mediated through Oxt in the mPFC. Oxt inputs to the mPFC result in the development, and support improvement of helping behaviors (Yamagishi et al., 2020; Li et al., 2021). Oxt injections in the mPFC (specifically the anterior cingulate area; ACA) of rhesus monkeys enhanced context dependent prosocial behaviors (Jiang and Platt, 2018). These suggest that Oxt can act throughout the dopaminergic VTA, serotonergic DR and PFC to regulate different levels of reward processing. Transcriptomic analysis of PVH-OXT neurons projecting to social reward brain regions are disrupted in an ASD mouse model and showed enrichment of ASD risk genes (Lewis et al., 2020). These reports suggest an exquisite role of Oxt in the development and maintenance of social reward memories, the perturbation of which results in ASD like phenotypes.

### Cognitive control module: Reproduction

Reproduction is a basic instinct crucial for survival of a species that can be defined as the process by which genetic material is transmitted from one generation to the next (Ishii and Touhara, 2019). Reproduction includes sexual activity, pregnancy, parturition, and lactation. Sexual activity in rodents follows mainly three steps: approach, investigation and mating. Approach and investigation results in the activation of olfactory and vomeronasal pathways. In male mice, pheromone signals from female mice are received in the AOB area and transmitted to the medial amygdala (MEA). The MEA inputs are then passed to the regulatory centers of reproduction in the hypothalamus- the preoptic area, the ventromedial hypothalamus (VMH) and the ventral premammilary nucleus (PMv). From the hypothalamus, signals are transmitted to the ventral midbrain followed by brainstem areas and the spinal cord that results in erection and ejaculation. In female mice, estrogen acts at the VMH and PAG, which is connected to the midbrain reticular nucleus (MRN) and then further through the reticulospinal tract to the spinal cord, that results in lordosis (Hashikawa et al., 2016).

Oxt acts at multiple brain areas to regulate different aspects of reproduction. During pregnancy and parturition, the role of hypothalamic release of Oxt is well discussed (Veening et al., 2010). Hypothalamic Oxt release is also important for lactation (Perkinson et al., 2021). Oxt fibers reach the MEA, multiple regions of the pre-optic area- medial preoptic area (MPO), medial preoptic nucleus (MPN), posterodorsal preoptic nucleus (PD), periventricular hypothalamic nucleus, preoptic part (PVpo), anteroventral preoptic nucleus (AVP) as well as the VMH and PMv. The MEA is also enriched with OxtRs. Oxt in the MEA alters sex preferences in male mice and is required to discriminate female mice from other male mice as well as female scent from male scent (Yao et al., 2017). It is also shown that Oxt is required in males for sexual experience based long term changes in MEA activity (Li et al., 2017). Oxt also plays a relevant role in regulating sexual behaviors through its actions in the preoptic area. OxtRs are expressed in the MPO, an area that mediates copulatory behaviors, with a sexual dimorphism of more OxtRs in males than females (Sharma et al., 2019). A further study from our lab identified sexual dimorphism in the anteroventral periventricular nucleus (AVPV), an area situated very close to the MPO (Newmaster et al., 2020). Intracerebral injections of Oxt in the MPO resulted in increased sexual behavior in male rats (Gil et al., 2011) and increased sexual receptivity in female rats (Caldwell et al., 1989). Moreover, Oxt in the MPO of monogamous mandarin voles resulted in better paternal care and OxtR antagonists reduced the total duration of paternal care as well as increased the latency to initiate paternal care (Yuan et al., 2019). All these reports suggest a major role for Oxt in the MPO in sexual and caregiving behaviors. The VMH is an estrogen receptor enriched area known for reproductive and metabolic regulation in female mice (Correa et al., 2015). Oxt mRNA was found to be higher in the VMH during certain stages of the estrous cycle in female rats suggesting a potential role of Oxt in luteinizing hormone release (Bale et al., 1995).

In mice, Oxt from the PVH to VTA induces erection (Melis et al., 2007). During pregnancy, increased Oxt binding to OxtRs was found in the SON, PVH and MPO (Bealer et al., 2006). A blockade in the binding of Oxt to OxtRs resulted in reduced milk yields upon suckling in rats and in marmosets (Lipschitz et al., 2003; Taylor and French, 2015). There is clinical evidence also suggesting a potential treatment possibility with Oxt for fertility and reproductive disorders. Oxt levels increase during sexual arousal and ejaculation in both men and women (Cera et al., 2021). Deficiencies in the Oxt system result in impotence and decreased libido whereas increased Oxt enhanced sexual behaviors (Magon and Kalra, 2011). Overall, Oxt plays multiple roles in reproduction, by exerting effects on the peripheral system as a hormone and within the CNS as a neuromodulator.

## Conclusion

In this review, we addressed functional circuits in the brain and explained the specific areas at which Oxt can exert its effect to modulate particular behaviors. We explained the neural circuits responsible for nine different functions and at what levels Oxt can act within each circuit. Oxt neuronal fibers reach all the major brain areas involved in most of the functional circuits. An exception to this is the thalamic regions (VPM, VPL, LG), the major relay station for sensory circuits and pain circuits to the cortex, that neither receive projection fibers, nor have OxtRs. We were also able to identify cross talk between multiple functional circuits and illustrate how Oxt can modulate different behaviors by acting at one brain area. For example, the PAG mediates fight and attack responses as well as receiving pain information. Another region that receives both pain and visual inputs is the PB. Social reward and memory areas in the brain are also heavily interconnected with each other and Oxt exerts effects on most areas within these two circuits to impact social memory (Nardou et al., 2019). Thus, Oxt can exert its effects at multiple levels of a functional circuit and alter the responses of more than one circuit due to its modulatory effects on crosstalk nodes. We hope that our review linking Oxt projection areas with functional circuits helps further research to unveil underexplored areas where Oxt can exert specific behavioral effects.

## Author contributions

SM and YK conceptualized the manuscript. SM wrote the initial manuscript and figures and further developed the manuscript with RB. YK handled the funding and critically revised the manuscript. All authors contributed to the article and approved the submitted version.
